# Impact of Pre-Diagnosed Depressive Symptoms on Treatment Choice, Delay in Initiating Treatment, and Mortality Among Women Aged ≥65 Years with Breast Cancer

**DOI:** 10.3390/ijerph23030361

**Published:** 2026-03-12

**Authors:** David Gbogbo, Rima Tawk, Askal A. Ali, Carlos A. Reyes-Ortiz, Gebre-Egziabher Kiros

**Affiliations:** 1 College of Pharmacy & Pharmaceutical Sciences, Institute of Public Health, Florida A & M University, Tallahassee, FL 32307, USA; david1.gbogbo@famu.edu (D.G.); carlos.reyesortiz@famu.edu (C.A.R.-O.); 2 Economic Social & Administrative Pharmacy, College of Pharmacy & Pharmaceutical Sciences, Institute of Public Health, Florida A & M University, Tallahassee, FL 32307, USA; askal.ali@famu.edu

**Keywords:** depressive symptoms, breast cancer, treatment delay, survivorship, health disparities, SEER-MHOS

## Abstract

**Highlights:**

**Public health relevance—How does this work relate to a public health issue?**

- Globally, depression is highly undertreated and can be linked to several chronic conditions, including breast cancer (BC).
- Women with pre-existing depressive symptoms at breast cancer diagnosis have a higher mortality risk than women without pre-existing depressive symptoms.

**Public health significance—Why is this work of significance to public health?**

- BC and depression continue to be diseases with tremendous public health significance, adversely affecting functional status, health-related quality of life, care utilization, and increasing medical costs.
- Pre-diagnosed depressive symptoms affect both the type of treatment women with BC receive and their survival outcomes.

**Public health implications—What are the key implications or messages for practitioners, policy makers, and/or researchers in public health?**

- Hispanic individuals are more likely to report delays in BC treatment; we recommend that future studies look at the impact of prior depressive symptoms on delay in treatment among minorities.
- Physicians, oncologists, psychiatrists, and public health researchers should work together to develop better strategies for optimizing the quality of care and reducing the disparities in BC outcomes among women with depressive symptoms.

**Abstract:**

Studies that have sought to describe and account for pre-diagnosed depressive symptoms on BC treatment choice, delay in initiating treatment, and mortality have been inconsistent. The purpose of the study is to examine the association between pre-diagnosed depressive symptoms and their impact on breast cancer (BC) treatment, treatment delays, and mortality. We conducted a retrospective cohort study using the Surveillance, Epidemiology, and End Results–Medicare Health Outcomes Survey (SEER-MHOS) dataset among women aged 65 years and older diagnosed with BC. Among 3840 eligible patients, 28.1% had pre-diagnosed depressive symptoms. Patients with pre-diagnosed depressive symptoms who were diagnosed with early-stage BC were significantly more likely (OR = 1.52; 95% CI: 1.26–1.84) to undergo mastectomy or receive breast-conserving surgery (BCS) alone rather than BCS plus radiation therapy (RT) compared to patients who were not pre-diagnosed with depressive symptoms. Among patients with advanced-stage BC, pre-diagnosed depressive symptoms were not significantly associated with treatment type. Among Hispanic patients, pre-diagnosed depressive symptoms were associated with treatment delays. Overall, patients with pre-diagnosed depressive symptoms had a 16% increased adjusted risk of BC-related mortality compared to those who were not pre-diagnosed with depressive symptoms, and those with advanced-stage cancer had an 18% higher adjusted risk of death than early-stage BC. Conclusions: Overlooking depressive symptoms management prior to a breast cancer diagnosis may result in poorer survival outcomes. Early detection and consistent management of depression are critical for improving patient survival.

## 1. Introduction

Breast cancer (BC) is the most common malignancy diagnosed in women, affecting one in eight women [1,2]. According to the American Cancer Society estimates, in 2024, about 310,720 new cases of invasive breast cancer were diagnosed in women, 56,500 new cases of ductal carcinoma in situ (DCIS) were diagnosed, and 42,250 women died from breast cancer [3]. Although breast cancer (BC) survival has improved over the years due to individualized treatment and earlier detection [4], most cases are found among the older population, and it is estimated that by 2030, approximately 2.3 million cases of BC will be diagnosed, and about 67% of the increase will be among older women [5,6].

BC treatments continue to evolve, and the decision on the choice of treatment is greatly influenced by lymph vascular spread, histologic grade, hormone receptor status (estrogen-ER or progesterone-PR), human epidermal growth factor receptor 2 status (HER2), comorbidities, patient menopausal status, and age [7]. Studies have revealed that the older population is more likely to be diagnosed with comorbidities [8], less likely to be represented in clinical trials [9], have lower odds of receiving standard care [10], more likely to deviate from BC treatment guidelines [11,12], and experience a delay in initiating treatment [13]. Unfortunately, evidence suggests that women with depression are more likely to face treatment delays and have a reduced likelihood of receiving definitive treatment [14,15,16] and experience the worst survival [17]. The burden of depression is also reflected in it being the cause of early death in the United States [18]. Pratt and colleagues indicated that persons with anxiety or depression died 7.9 years earlier than other persons [19], and studies have also found that depression is related to increased mortality among BC patients [17,20]. Nevertheless, the impact of pre-diagnosed depressive symptoms on both patients’ and physicians’ decision-making processes when it comes to treatment choices, initiating treatment, and further, the overall impact on mortality, is an area that has not been fully explored. Studies that have sought to describe and account for pre-diagnosed depressive symptoms on BC treatment choice, delay in initiating treatment, and mortality have been inconsistent [14,17,20,21,22]. Hence, we sought to examine whether pre-diagnosed depressive symptoms are associated with local therapy decisions for early-stage and advanced-stage BC, delay in initiating treatment, and finally, their effect on mortality among older women represented in the Surveillance, Epidemiology and End Results–Medicare Health Outcomes Survey (SEER-MHOS) linked data resource.

## 2. Methods

### 2.1. Data Source

**Figure 1:**
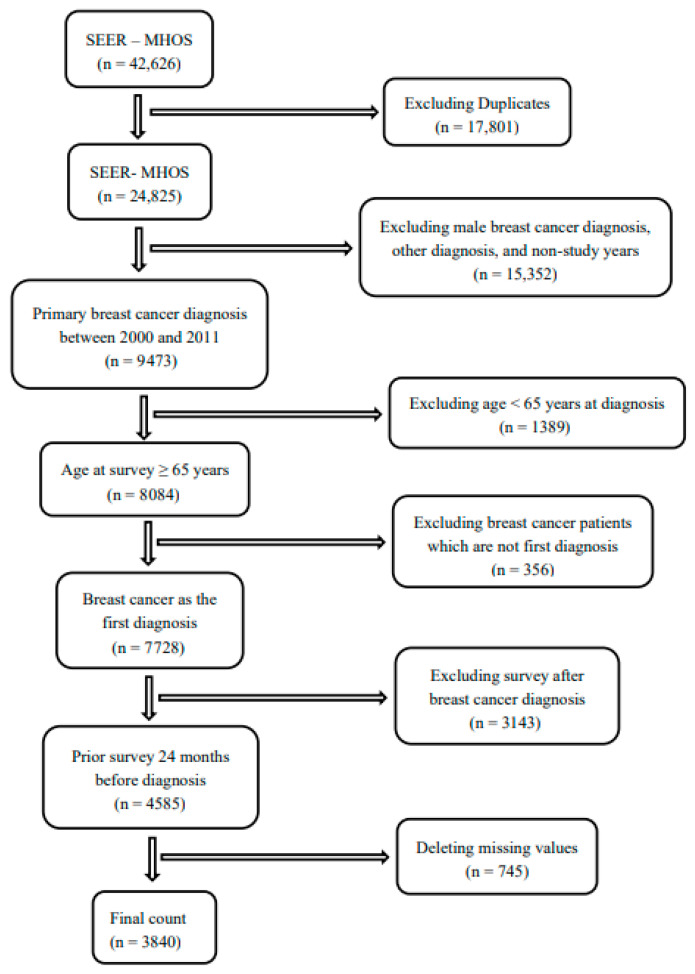
Flowchart of all breast cancer patients extracted from the SEER-MHOS data resource from 2000 to 2011.

The study is based on a retrospective cohort analysis of depressive symptoms and BC patients aged 65 and above located in the linked Surveillance, Epidemiology, and End Results—Medicare Health Outcomes Survey (SEER-MHOS) data resource. The SEER-MHOS linked data resource comes from the combined effort of the National Cancer Institute (NCI), the SEER registries, and the Centers for Medicare & Medicaid Services (CMS), which links data between three data sources: SEER clinical data, Medicare Part D claims, and MHOS data [23]. SEER-MHOS collects clinical, initial treatment information, demographic, self-reported socioeconomic, comorbid conditions, symptoms, and HRQOL, administered annually to randomly selected Medicare managed care beneficiaries [24,25]. Selected patients who are alive and remain in the same managed care plan receive follow-up surveys at 2 years [26]. This study was determined to be exempt from review by the Florida A&M University Institutional Review Board (064-23; 2066577-1). The study population consists of all women aged 65 years and older who were newly diagnosed with stage I-IV BC with or without pre-diagnosed depressive symptoms between 2000 and 2011 in the United States. Women whose first or primary diagnoses after the two-year survey were not BC were excluded (Figure 1).

### 2.2. Variables

#### 2.2.1. Predictor Variable

Depressive symptoms screener was assessed by 3 questions: “Two Weeks of Depression in Past Year (yes/no)”, “Depression Much of the Time in Past Year (yes/no)”, and “Depression Most of the Time for 2 Years (yes/no)”. Participants were determined to be at risk of depression if they answered “yes” to any of the questions. The utilization of this algorithm in identifying individuals with depression has been previously demonstrated in other studies [22,27,28].

#### 2.2.2. Outcome Variables

BC stage diagnoses were classified into either early or advanced stage using the breast-adjusted American Joint Committee on Cancer (AJCC) 6th tumor (T), lymph nodes (N), and metastasis (M) variables. In the dataset, BC treatment was limited to local therapy. It was classified into three levels: mastectomy, breast-conserving surgery (BCS), and breast-conserving surgery with radiation therapy (BCS and RT). Delay in treatment was computed by discounting the date of BC diagnosis from the date of first treatment. The threshold of 60 days was adopted because Iglay et al. found that an increase in mortality was associated with a 2- to 3-month delay in treatment [29]. BC-specific death was defined as death from BC whiles deaths from all other causes were censored. Information on months of survival from the date of diagnosis was provided in SEER. The last date of the follow-up for this cohort was 31 December 2011.

#### 2.2.3. Covariates

Sociodemographic characteristics included age at diagnosis (65–74 years, 75–84 years, or 85+ years), marital status (married or unmarried), race (White, Black, or Hispanic), and education (below high school, high school graduate, or college graduate). Clinical characteristics included tumor grade (well-differentiated tumor, moderately differentiated tumor, or poorly differentiated/undifferentiated), hormone receptor status (ER- or PR-positive, ER- and PR-negative). Comorbidity index was created for the following chronic conditions: “Emphysema, Asthma, or COPD”, “Hypertension”, “Diabetes”, “Angina Pectoris/Coronary Artery Disease”, “Congestive Heart Failure”, “Myocardial Infarction”, “Other Heart Conditions”, “Stroke”, “Crohn’s Disease, Ulcerative Colitis, or Inflammatory Bowel”, “Sciatic”, “Arthritis of Hand/Wrist”, and “Arthritis of Hip/Knee”. This index was re-coded to generate 3 categories (0, 1–2, and 3+).

### 2.3. Data Analysis

Analyses were conducted separately for early and advanced-stage BC patients. The Pearson chi-square test of independence was used to assess independence between the women with and without pre-diagnosed depressive symptoms. Multivariable multinomial logistic regression was used to assess associations between pre-diagnosed depressive symptoms and receipt of treatment for patients with early-stage BC. Multiple logistic regression analysis was used to examine the association between pre-diagnosed depressive symptoms and receipt of treatment for women with advanced-stage BC and delay in treatment, respectively. Finally Cox proportional hazards model was used to analyze the effect of pre-diagnosed depressive symptoms on BC mortality. All statistical analyses were conducted with SAS software, version 9.4 (SAS Institute, Cary, NC, USA).

## 3. Results

### 3.1. Participant Characteristics

We identified 3840 patients with BC diagnosis who had completed the Health Outcome Survey (HOS) 24 months before their diagnosis. Among them, 1078 (28.1%) reported pre-diagnosed depressive symptoms before their BC diagnosis. Regarding the stage of BC at diagnosis, 3205 patients (83.5%) were diagnosed at an early stage, while 635 (16.5%) had an advanced-stage disease. In terms of initial treatment, 1199 patients (31.2%) underwent mastectomy, 1057 (27.6%) received only breast-conserving surgery (BCS), and 1584 (41.2%) underwent breast-conserving surgery with radiation therapy (BCS and RT). Additionally, 3605 patients (95.5%) received treatment within 60 days of diagnosis, while 170 (4.5%) started treatment after 60 days. By the end of the study, 930 patients (24.2%) had died, while 2910 (75.8%) were alive (Table 1).

### 3.2. Association Between Pre-Diagnosed Depressive Symptoms and Treatment Received for Early Stage

Among patients with early-stage BC, 27.9% (n = 894) reported depressive symptoms 24 months before their BC diagnosis. The unadjusted odds ratio showed that early-stage BC patients with pre-diagnosed depressive symptoms had 25% higher odds of undergoing mastectomy and 52% higher odds of receiving only breast-conserving surgery (BCS) (OR = 1.52; 95% CI: 1.26–1.84) compared to those who underwent BCS plus radiation therapy (RT).

After adjusting for sociodemographic and clinical covariates, early-stage BC patients who reported depressive symptoms had 33% higher odds of undergoing mastectomy (OR = 1.33; 95% CI: 1.04–1.71) and 58% higher odds of receiving only BCS (OR = 1.58; 95% CI: 1.21–2.07) compared to those who received BCS plus RT.

Additionally, patients with poorly differentiated or undifferentiated tumor grades had 39% higher odds of undergoing mastectomy (OR = 1.39; 95% CI: 1.00–1.92). Patients with negative estrogen receptor (ER) and progesterone receptor (PR) status had 51% higher odds of undergoing mastectomy compared to those who received BCS plus RT (OR = 1.51; 95% CI: 1.17–1.92).

Patients aged 85 and older were five times more likely to undergo mastectomy and nine times more likely to receive only BCS compared to those who underwent BCS plus RT. Similarly, patients aged 75–84 had 39% and 52% higher odds of undergoing mastectomy and BCS, respectively.

### 3.3. Association Between Pre-Diagnosed Depressive Symptoms and Treatment Received for Advanced Stage

Among patients with advanced-stage BC, 29% (n = 184) were at risk of depressive symptoms 24 months before their BC diagnosis (Table 1). Results from both unadjusted and adjusted odds ratios indicated that pre-diagnosed depressive symptoms were not significantly associated with the type of treatment received (Table 2).

However, advanced-stage BC patients with pre-diagnosed depressive symptoms who were unmarried were twice as likely to undergo mastectomy and four times more likely to receive only breast-conserving surgery (BCS) compared to those who received BCS plus radiation therapy (RT). Additionally, patients with less than a high school education were three times more likely to undergo mastectomy and twice as likely to receive only BCS compared to those who received BCS plus RT.

### 3.4. Association Between Pre-Diagnosed Depressive Symptoms and Delay in Treatment

Among patients who received treatment within 60 days of their BC diagnosis, 27.7% (n = 1001) were diagnosed with depressive symptoms. Among those who received treatment after 60 days, 32.4% (n = 55) were diagnosed with depressive symptoms (Table 1). Results indicated that pre-diagnosed depressive symptoms were not significantly associated with treatment delay in both unadjusted and adjusted models (Table 3). However, in the adjusted model, Hispanics with pre-diagnosed depressive symptoms were three times more likely to experience a treatment delay of more than 60 days compared to Whites (OR = 3.34; 95% CI: 1.95–5.73).

### 3.5. Association Between Pre-Diagnosed Depressive Symptoms and BC Mortality

The adjusted risk of BC (BC)-related mortality was 16% higher in patients with pre-diagnosed depressive symptoms compared to those without (HR = 1.16; 95% CI: 1.05–1.28) (Table 4). Additionally, the adjusted risk of BC-related mortality was 18% higher in patients with advanced-stage BC compared to those with early-stage disease (HR = 1.18; 95% CI: 1.04–1.34). Compared to patients aged 65–74, the adjusted hazard ratio (HR) for BC-related death was 25% higher among those aged 75–84 (HR = 1.25; 95% CI: 1.13–1.37) and twice as high among patients aged 85 and older (HR = 2.05; 95% CI: 1.78–2.35). No association was observed between education level and mortality. Additionally, the HR of death was 24% higher in patients with three or more comorbidities compared to those without any comorbidities (Table 4).

## 4. Discussion

Patients with pre-diagnosed depressive symptoms who were diagnosed with early-stage BC were significantly more likely to undergo mastectomy or receive breast-conserving surgery (BCS) alone rather than the recommended BCS plus radiation therapy (RT) compared to patients who were not pre-diagnosed with depressive symptoms. This relationship remained significant after adjusting for sociodemographic and clinical characteristics. Our findings are consistent with previous studies [17,22]. However, pre-diagnosed depressive symptoms were not associated with the receipt of treatment among advanced-stage BC patients. Furthermore, a prior diagnosis of depressive symptoms was not associated with delays in the treatment of BC, similar to the results found by Iglay et al. [29] and Burgess et al. [30]. However, our study found that Hispanic individuals were about three times more likely to experience more than 60 days of delay in treatment compared to White individuals. Structural, linguistic, and access-related factors could contribute to treatment delays among Hispanic patients. Hispanic adults face financial limitations and challenging socioeconomic conditions, such as longer distances to access care, language barriers, lack of health insurance, and limited healthcare access. They tend to be diagnosed at later BC stages, possibly due to lower mammography screening rates, resulting in delays in timely follow-up oncologic care [31]. Further, women with pre-diagnosed depressive symptoms at BC diagnosis have a higher mortality hazard than women without pre-diagnosed depressive symptoms. Many studies have shown that depression is associated with increased mortality [17,32,33].

The impact of pre-diagnosed depressive symptoms on the treatment choice is not well understood. Depression has been found to decrease treatment compliance [34,35]. The relationship between depression and care-seeking behavior is complex, and the relationship can differ depending on the severity [36]. Depression is known to be associated with a perceived low level of self-efficacy [37]. Patients with depression often lose interest and have difficulty finding the motivation to conduct daily activities [29,38]. They are less likely to advocate for their health, to participate in BC screening, and to adhere to cancer treatment [36]. Depression can also increase the mortality rate of BC patients in numerous ways. Factors such as lifestyle, behavioral factors, and biological factors have been seen to influence this relationship. First, depressed patients are more prone to unhealthy lifestyle factors, such as smoking, drinking, obesity, and insomnia, which may increase the risk of mortality and cardiovascular disease [39]. Second, the lower likelihood of advocating for one’s health or care-seeking, BC screening, nonadherence to treatment regimens, suicidal thoughts, and lengthened hospitalization is higher among depressive patients [35,40,41], and these are linked to lower survival. Our findings should be interpreted with a few caveats. While the SEER-MHOS data resource allowed us to examine the effects of pre-diagnosed depressive symptoms on the stage of BC, the depressive diagnoses were self-reported and may indicate an overestimation or underestimation of depressive symptoms [22]. The MHOS depression screener differs from clinically diagnosed depression and thus could lead to a possible misclassification bias due to the nature of self-reported data. Another limitation is that our dataset does not capture the receipt of psychological treatment or support for patients who reported pre-diagnosed depressive symptoms after the BC diagnosis. In addition, BC treatment information was only available for local therapy. Systemic treatments such as chemotherapy, endocrine therapy, or targeted therapy were not captured in our dataset and thus could not be considered. The exclusion of systemic therapies may impact our treatment and survival outcomes findings. The study is novel in its ability to examine the relationship between treatment and self-reported depressive symptoms before patients are aware of their BC diagnosis. The public health implications highlight the importance of prevention efforts and counseling to support mental health and prevent the initial emergence of depressive symptoms among older women. More health education efforts should be carried out to render doctors, patients, and their families aware of the importance of mental well-being. According to Buscariollo and colleagues, understanding this mechanism may provide an opportunity to develop strategies for enhancing supportive resources and physician communication during counseling which may optimize the quality of care and reduce the disparities in BC outcomes among individuals with mental illness in general [22]. More research is needed to further explore potential mechanisms by which depressive symptoms may influence treatment decisions. Further, even though depressive symptoms was not associated with delay in treatment, our findings indicated that minority women are more likely to report delays in treatment, we recommend that future studies should look at the impact of prior depressive symptoms on delay in treatment among minority women with BC.

## 5. Conclusions

Pre-diagnosed depressive symptoms affect the type of treatment women with BC receive. Older women with pre-diagnosed depressive symptoms did not report delays in BC patients’ initiation to care, except for Hispanic individuals. However, women with pre-diagnosed depressive symptoms at breast cancer diagnosis have a higher mortality hazard than women without pre-diagnosed depressive symptoms. The role of physicians is vital in detecting depression among BC diagnosed patients. Hence, they should work together with oncologists, pharmacists, and psychiatrists to develop better targeted treatment options in the coordination of care and treatment for these patients.

## Figures and Tables

**Table 1:** Patients’ characteristics for women diagnosed with breast cancer.

Characteristics			Pre-Diagnosed Depressive Symptoms				
	Number of Patients		No		Yes		*p*-Value ^#^
	N	%	N	%	N	%	
			2762	71.93	1078	28.07	
Age at diagnosis, Mean (SD)	69 (7.2)						0.9128
65–74 years	1394	46.14	992	71.16	402	28.84	
75–84 years	1159	38.36	832	71.79	327	28.21	
85+ years	468	15.49	337	72.01	131	27.99	
Race							<0.0001
White	2908	82.52	2145	73.76	763	26.24	
Black	333	9.45	205	61.56	128	38.44	
Hispanic	283	8.03	172	60.78	111	39.22	
Marital status							<0.0001
Married	1796	47.63	1393	77.56	403	22.44	
Unmarried	1975	52.37	1315	66.58	660	33.42	
Education							<0.0001
Below high school	947	25.12	594	62.72	353	37.28	
High school graduate	1419	37.64	1043	73.50	376	26.50	
College education	1404	37.24	1078	76.78	326	23.22	
No. of comorbidity							<0.0001
0	907	23.70	774	85.34	133	14.66	
1–2	1741	45.49	1286	73.87	455	26.13	
3+	1179	30.81	694	58.86	485	41.14	
Breast cancer stage							0.5792
Early stage	3205	83.46	2311	72.11	894	27.89	
Advanced stage	635	16.54	451	71.02	184	28.98	
Tumor grade							0.3282
Well-differentiated	1010	28.12	737	72.97	273	27.03	
Moderately differentiated	1676	46.66	1217	72.61	459	27.39	
Poorly differentiated/undifferentiated	906	25.22	636	70.20	270	29.80	
Hormone receptor status							0.6500
ER- or PR-positive	2465	70.57	1775	72.01	690	27.99	
ER- and PR-negative	1028	29.43	748	72.76	280	27.24	
Treatment received							0.0002
Mastectomy	1199	31.22	854	71.23	345	28.77	
Breast-conserving surgery	1057	27.53	717	67.83	340	32.17	
Breast-conserving surgery and radiation	1584	41.25	1191	75.19	393	24.81	
Delay in treatment							0.1930
<60 days	3605	95.50	2604	72.23	1001	27.77	
≥60 days	170	4.50	115	67.65	55	32.35	
Vital status							0.0034
Dead	930	24.22	634	68.17	296	31.83	
Alive	2910	75.78	2128	73.13	782	26.87	

^#^ A Chi-square test is used to measure bivariate associations.

**Table 2:** Associations between pre-diagnosed depressive symptoms and treatment received.

Stage		Treatment Received	Unadjusted		Adjusted ^#^	
			OR	95% CI	OR	95% CI
Early	Model 1	Mastectomy vs. BCS and RT	1.25 *	1.04, 1.51		
		BCS only vs. BCS and RT	1.52 *	1.26, 1.84		
	Model 2	Clinical Characteristics				
		Mastectomy vs. BCS and RT			1.22	1.00, 1.50
		BCS only vs. BCS and RT			1.41 *	1.41, 1.74
	Model 3	Sociodemographic Characteristics				
		Mastectomy vs. BCS and RT			1.24	0.99, 1.56
		BCS only vs. BCS and RT			1.56 *	1.23, 1.98
	Model 4	All Covariates				
		Mastectomy vs. BCS and RT			1.33 *	1.04, 1.71
		BCS only vs. BCS and RT			1.58 *	1.21, 2.07
Advanced	Model 1	Mastectomy vs. BCS and RT	0.95	0.58, 1.56		
		BCS only vs. BCS and RT	1.01	0.62, 1.64		
	Model 2	Clinical Characteristics				
		Mastectomy vs. BCS and RT			1.00	0.57, 1.74
		BCS only vs. BCS and RT			1.16	0.66, 2.03
	Model 3	Sociodemographic Characteristics				
		Mastectomy vs. BCS and RT			0.82	0.45, 1.48
		BCS only vs. BCS and RT			0.74	0.41, 1.35
	Model 4	All Covariates				
		Mastectomy vs. BCS and RT			0.91	0.46, 1.81
		BCS only vs. BCS and RT			0.92	0.45, 1.84

* Significant at α < 0.05. ^#^ Adjusted for age at diagnosis, race and ethnicity, marital status, education, number of comorbidities, hormone receptor status, and tumor grade.

**Table 3:** Associations between pre-diagnosed depressive symptoms and delay in treatment.

	Outcomes	Unadjusted		Adjusted ^#^	
		OR	95% CI	OR	95% CI
Model 1	≥60 days vs. <60 days	1.24	0.90, 1.73		
Model 2	Clinical Characteristics				
	≥60 days vs. <60 days			1.20	0.83, 1.74
Model 3	Sociodemographic Characteristics				
	≥60 days vs. <60 days			1.26	0.85, 1.86
Model 4	All Covariates				
	≥60 days vs. <60 days			1.13	0.73, 1.76

^#^ Adjusted for age at diagnosis, race and ethnicity, marital status, education, number of comorbidities, hormone receptor status, and tumor grade.

**Table 4:** Cox proportional hazard models for mortality for breast cancer patients by sociodemographic and clinical characteristics.

Characteristics	Unadjusted		Adjusted ^#^	
	HR	95% CI	HR	95% CI
Pre-diagnosed depressive symptoms				
No	1		1	
Yes	1.20 *	1.13, 1.29	1.16 *	1.05, 1.28
Breast cancer stage				
Early stage			1	
Advance stage			1.18 *	1.04, 1.34
Tumor grade				
Well-differentiated			1	
Moderately differentiated			1.00	0.90, 1.11
Poorly differentiated/undifferentiated			0.99	0.87, 1.13
Hormone receptor status				
ER- or PR-positive			1	
ER- and PR-negative			0.94	0.85, 1.04
Treatment received				
Mastectomy			0.97	0.88, 1.08
Breast-conserving surgery only			1.16 *	1.03, 1.30
Breast-conserving surgery and radiation			1	
Age at diagnoses				
65–74 years			1	
75–84 years			1.25 *	1.13, 1.37
85+ years			2.05 *	1.78, 2.35
Race				
White			1	
Black			1.12	0.96, 1.32
Hispanic			1.02	0.87, 1.20
Marital status				
Married				1
Unmarried			1.04	0.95, 1.14
Education				
Below high school			1.04	0.92, 1.16
High school graduate			1	
College education			1.06	0.96, 1.16
No. of comorbidities				
0			1	
1–2			1.05	0.94, 1.17
3+			1.24 *	1.10, 1.39

* Significant at α < 0.05. ^#^ Adjusted for breast cancer stage, tumor grade, hormone receptor status, treatment received and age at diagnosis, race and ethnicity, marital status, education, and number of comorbidities.

## Data Availability

This study used data from the Surveillance, Epidemiology, and End Results (SEER)–Medicare Health Outcomes Survey (MHOS) linked data resource. The original contributions presented in this study are included in the article. The datasets used and/or analyzed during the current study are available from the corresponding author on reasonable request (Rima Tawk: rima.tawk@famu.edu). Restrictions apply to the availability of these data.
